# *MET* or *NRAS* amplification is an acquired resistance mechanism to the third-generation EGFR inhibitor naquotinib

**DOI:** 10.1038/s41598-018-20326-z

**Published:** 2018-01-31

**Authors:** Kiichiro Ninomiya, Kadoaki Ohashi, Go Makimoto, Shuta Tomida, Hisao Higo, Hiroe Kayatani, Takashi Ninomiya, Toshio Kubo, Eiki Ichihara, Katsuyuki Hotta, Masahiro Tabata, Yoshinobu Maeda, Katsuyuki Kiura

**Affiliations:** 1 Department of Hematology, Oncology and Respiratory Medicine, Okayama University Graduate School of Medicine, Dentistry and Pharmaceutical Sciences, Okayama, Japan; 2 Department of Respiratory Medicine, Okayama University Hospital, Okayama, Japan; 3 Department of Biobank, Okayama University Graduate School of Medicine, Dentistry and Pharmaceutical Sciences, Okayama, Japan; 4 Center for Clinical Oncology, Okayama University Hospital, Okayama, Japan; 5 Center for Innovative Clinical Medicine, Okayama University Hospital, Okayama, Japan

## Abstract

As a third-generation epidermal growth factor receptor (EGFR) tyrosine kinase inhibitor (TKI), osimeritnib is the standard treatment for patients with non-small cell lung cancer harboring the *EGFR* T790M mutation; however, acquired resistance inevitably develops. Therefore, a next-generation treatment strategy is warranted in the osimertinib era. We investigated the mechanism of resistance to a novel EGFR-TKI, naquotinib, with the goal of developing a novel treatment strategy. We established multiple naquotinib-resistant cell lines or osimertinib-resistant cells, two of which were derived from EGFR-TKI-naïve cells; the others were derived from gefitinib- or afatinib-resistant cells harboring *EGFR* T790M. We comprehensively analyzed the RNA kinome sequence, but no universal gene alterations were detected in naquotinib-resistant cells. Neuroblastoma RAS viral oncogene homolog (*NRAS*) amplification was detected in naquotinib-resistant cells derived from gefitinib-resistant cells. The combination therapy of MEK inhibitors and naquotinib exhibited a highly beneficial effect in resistant cells with *NRAS* amplification, but the combination of MEK inhibitors and osimertinib had limited effects on naquotinib-resistant cells. Moreover, the combination of MEK inhibitors and naquotinib inhibited the growth of osimertinib-resistant cells, while the combination of MEK inhibitors and osimertinib had little effect on osimertinib-resistant cells. Clinical assessment of this novel combination (MEK inhibitors and naquotinib) is worth considering in osimertinib-resistant lung tumors.

## Introduction

The discovery of somatic mutations in epidermal growth factor receptor (*EGFR*) and EGFR tyrosine kinase inhibitors (TKIs) has dramatically improved the survival of patients with advanced non-small cell lung cancer (NSCLC) harboring *EGFR* mutations^1–3^. However, lung tumors inevitably acquire resistance to first- or second-generation EGFR-TKIs around 12 months^4–6^. Therefore, it is very important to clarify the mechanisms of resistance and establish corresponding treatment strategies. Multiple studies have revealed that *EGFR* T790M is the most frequent mechanism of resistance.

To overcome the *EGFR* T790M mutation, third-generation (3^rd^-gen) EGFR-TKIs such as osimertinib and nazartinib have been developed. Currently, osimertinib has been clinically approved for patients with lung tumors harboring *EGFR* T90M^7^. 3^rd^-gen EGFR-TKIs effectively inhibit both resistant and sensitive *EGFR* mutations (e.g., *EGFR* L858R and *EGFR* 15 bp DEL)^8–10^, while exhibiting less sensitivity to wild-type EGFR and resulting in less skin rush and diarrhea^7^. Naquotinib, a novel 3^rd^-gen EGFR-TKI, showed a promising effect (response rate 64%) in a phase 2 trial in Japanese patients with *EGFR* T790M-positive lung cancer^11^; however, its clinical development was discontinued for unpublished reasons.

Unfortunately, acquired resistance is unavoidable for these 3^rd^-gen EGFR-TKIs. The median progression-free survival (mPFS) in *EGFR* T790M-positive lung tumors is approximately 10 months^7,12^. The mPFS is unprecedented but is still unsatisfactory for patients and clinicians. Several mechanisms of resistance to 3^rd^-gen EGFR-TKIs, such as the resistant *EGFR* C797S mutation, RAS/ERK activation, YES1 activation, HER2 activation, and *MET* amplification, have been reported in preclinical and clinical studies^13–17^. The inhibitory profile of each 3^rd^-gen EGFR-TKI may vary, and each mechanism of resistance has not been fully elucidated. Therefore, it is necessary to explore each mechanism of resistance and develop new treatment strategies to overcome resistance to 3^rd^-gen EGFR-TKIs.

To explore the mechanism of resistance to naquotinib, we established multiple naquotinib-resistant lung cancer cell lines from EGFR-TKI-naïve or EGFR-TKI pre-exposed resistant cells, and we performed a comprehensive analysis, which included next-generation sequencing. Furthermore, we tested whether naquotinib was effective against osimertinib-resistant lung cancer cells.

## Materials and Methods

### Cell lines, cell culture, and reagents

PC-9 cells (*EGFR* Ex19 del E746_A750) were purchased from the European Collection of Cell Cultures in 2014. RPC-9 cells (gefitinib-resistant; *EGFR* Ex19 del E746_A750 and Ex20 T790M) were established from a parental PC-9 cell line in our laboratory^18^. HCC827 cells (*EGFR* Ex19 del E746_A750)^5^ and PC-9/BRc1 cells (afatinib-resistant; *EGFR* Ex19 del E746_A750 and Ex20 T790M)^19^ were kindly provided by Dr. William Pao (Vanderbilt University, Nashville, TN, USA). Cells were cultured in RPMI-1640 medium (Sigma-Aldrich, St. Louis, MO, USA) supplemented with 10% heat-inactivated fetal bovine serum and 1% penicillin/streptomycin in a tissue culture incubator at 37 °C under 5% CO_2_.

Naquotinib was provided by Astellas Pharma Inc. (Tokyo, Japan) under a material transfer agreement. Gefitinib, afatinib, osimertinib, crizotinib, SGX-523, selumetinib, and trametinib were purchased from Selleck Chemicals (Houston, TX, USA). UNC569 was purchased from Merck Millipore (Billerica, MD, USA). All compounds were dissolved in dimethyl sulfoxide for *in vitro* studies.

Growth inhibition was measured using a modified 3-(4,5-dimethylthiazol-2-yl)-2,5-diphenyltetrazolium bromide (MTT) assay^20^. Briefly, cells were plated onto 96-well plates at a density of 2,000–3,000 per well and continuously exposed to each drug for 96 h.

### Antibodies, immunoblotting, and receptor tyrosine kinase array

The following antibodies were obtained from Cell Signaling Technology (Danvers, MA, USA): phospho-EGFR, EGFR, phospho-MET, phospho-ERK, ERK, phospho-AKT, AKT, E-cadherin, vimentin, GAPDH, and horseradish peroxidase (HRP)-conjugated anti-rabbit. MET and NRAS antibodies were purchased from Santa Cruz Biotechnology (Dallas, TX, USA). For immunoblotting, cells were harvested, washed in phosphate-buffered saline, and lysed in radioimmunoprecipitation assay buffer (1% Triton X-100, 0.1% sodium dodecyl sulfate [SDS], 50 mM Tris-HCl, pH 7.4, 150 mM NaCl, 1 mM EDTA, 1 mM EGTA, 10 mM β-glycerol-phosphate, 10 mM NaF, and 1 mM sodium orthovanadate) containing a protease inhibitor tablet (Roche Applied Sciences, Penzberg, Germany). Lysates were subjected to SDS-polyacrylamide gel electrophoresis (PAGE), proteins were transferred to membranes and incubated with the indicated antibodies, and detected using enhanced chemiluminescence plus reagents (GE Healthcare Biosciences, Pittsburgh, PA, USA). A Phospho-Receptor Tyrosine Kinase (RTK) Array Kit was purchased from R&D Systems (Minneapolis, MN, USA) and used according to the manufacturer’s recommendations. Bands and dots were detected using an ImageQuant LAS-4000 imager (GE Healthcare Biosciences).

### Immunohistochemistry

Formalin-fixed, paraffin-embedded tissue blocks were cut to a thickness of 5 μm, placed on glass slides, and deparaffinized in d-limonene and graded alcohol. The antigen was incubated in 1 mM EDTA buffer (pH 8.0) for 10 min in a 95 °C water bath. Sections were then blocked for endogenous peroxidase with 3% hydrogen peroxide in methanol for 10 min. Slides were rinsed with Tris-buffered saline containing 0.1% Tween 20 and blocked with normal goat serum for 60 min. Sections were incubated with anti-MET antibody overnight at 4 °C and amplified using biotinylated anti-rabbit antibodies and avidin-biotinylated HRP conjugate for 20 min (LSABTM 2 Kit; DakoCytomation, Glostrup, Denmark). Sections were then reacted with 3,3-diaminobenzidine and counterstained with hematoxylin.

### Quantitative polymerase chain reaction (qPCR) and fluorescence *in situ* hybridization (FISH)

DNA was extracted from cells using a QIAamp DNA Mini Kit (Qiagen, Venlo, Netherlands) according to the manufacturer’s protocol. The *MET* and *NRAS* copy number gains were determined by qPCR analysis of DNA using TaqMan probes and primers (details are provided in Supplementary Table 1A). PCR amplification was performed on a LightCycler Real-Time PCR System (Roche Applied Science), and gene dosage was calculated using a standard curve. The copy number ratio of the target gene to *GAPDH* was calculated.

FISH analysis of *MET* and *CEP7* was performed by SRL Inc. (Tokyo, Japan) using *MET* SpectrumRed and *CEP7* SpectrumGreen probes, respectively.

### Targeted RNA sequencing

RNA was extracted from each cell line using a QIAamp DNA Mini Kit (Qiagen) or an RNeasy Mini Kit (Qiagen) according to the manufacturer’s protocol. RNA (1,500 pg per cell line) was used for targeted RNA sequencing using a SureSelect RNA Human Kinome Kit (Agilent Technologies, Santa Clara, CA, USA) targeting 612 genes, including 517 protein kinases. Sequencing was performed on a MiSeq Sequencing System via a V2 Reagent Kit (Illumina, San Diego, CA, USA). Sequencing data were analyzed using the CLC Genomics Workbench (CLC bio, Aarhus, Denmark). RNA samples from each cell line were analyzed twice and averaged.

### Direct sequencing of *EGFR* and *NRAS*

*EGFR* exons 19 and 20 and *NRAS* exons 2 and 3 were amplified from genomic DNA. PCR products were processed using the BigDye Terminator Cycle Sequencing Kit v3.1 (Applied Biosystems, Foster City, CA, USA) according to the manufacturer’s protocol, and analyzed in both the sense and antisense directions for the presence of mutations on an ABI 3100 sequencer (Applied Biosystems). The primer sequences are provided in Supplementary Table 1B.

The analysis of *NRAS* and *KRAS* mutations was performed by BML Inc. (Tokyo, Japan) according to the reverse sequence-specific oligonucleotide with PCR method.

### RAS activation assay

RPC-9, and naquotinib or osimertinib-resistant cancer cells were serum starved overnight and supplemented with 1 μmol/L naquotinib for 4 h. RAS activity was measured using the Ras-binding domain of Raf-1 to pull down active Ras according to the manufacturer’s protocol (Cell BioLabs, San Diego, CA, USA). Following separation by SDS-PAGE, proteins were transferred to membranes and probed with an anti-NRAS antibody.

### Xenograft models

Female BALB/c nu/nu mice (7 weeks old) were purchased from Charles River Laboratories, Japan. All mice were provided sterilized food and water, and were housed in a barrier facility under a 12-h light/dark cycle. Cancer cells (2 × 10^6^) were injected subcutaneously into the backs on both sides of the mice. When the average tumor volume reached approximately 100 mm^3^, the mice were randomly assigned to one of four groups (3–4 mice per group) that received vehicle, naquotinib (50 mg/kg/day), crizotinib (25 mg/kg/day), or a combination of the two drugs for the same duration. The vehicle and drugs were administered once daily, five times per week by gavage. Tumor volume (width^2^ × length/2) was determined periodically. Statistical data were analyzed on day 28. All experiments involving animals were performed under the auspices of the Institutional Animal Care and Research Advisory Committee at the Department of Animal Resources, Okayama University Advanced Science Research. The experiments were performed under the Policy on the Care and Use of the Laboratory Animals, Okayama University and Fundamental Guidelines for Proper Conduct of Animal Experiment and Related Activities in Academic Research Institutions, Ministry of Education, Culture, Sports, Science and Technology-Japan. The experimental protocol was approved by the Animal Care and Use Committee, Okayama University, Okayama, Japan (OKU-2017084).

### Statistical analysis

Statistical analysis was performed using the JMP 13 program (SAS Institute, Cary, NC, USA). Group differences were compared using the two-tailed paired Student’s *t*-test. A P-value of < 0.05 was considered significant.

## Results

### Heterogeneous gene expression of naquotinib resistance in a cell line-based model

First, to explore the mechanism of resistance to naquotinib, we established naquotinib-resistant lung cancer cells using a cell line-based model. The following cell lines were examined: 1. EGFR-TKI-naïve PC-9 cells harboring the *EGFR* exon 19del, 2. acquired gefitinib-resistant RPC-9 cells harboring the *EGFR* exon 19del and T790M mutations, and 3. acquired afatinib-resistant PC-9/BRc1 cells harboring the *EGFR* exon 19del and T790M mutations. As expected, the 3^rd^-gen EGFR-TKI naquotinib effectively inhibited cell proliferation and the EGFR signaling pathway in each cell line (Supplementary Fig. 1A and B). Using a dose-escalation method (exposing naquotinib from 0.01 to 1.0 µmol/L), we established resistant cell lines from each parental cell line, which were designated PC-9/NaqR, RPC-9/NaqR, and PC-9/BRc1/NaqR, respectively (Supplementary Fig. 2A), and were confirmed to be identical (Supplementary Fig. 2C). The times to establishment of the naquotinib-resistant lung cancer cell lines were 5.2, 7.8, and 6.4 months in PC-9, RPC-9, and PC-9/BRc1 cells, respectively (Supplementary Fig. 2B).

**Figure 1:**
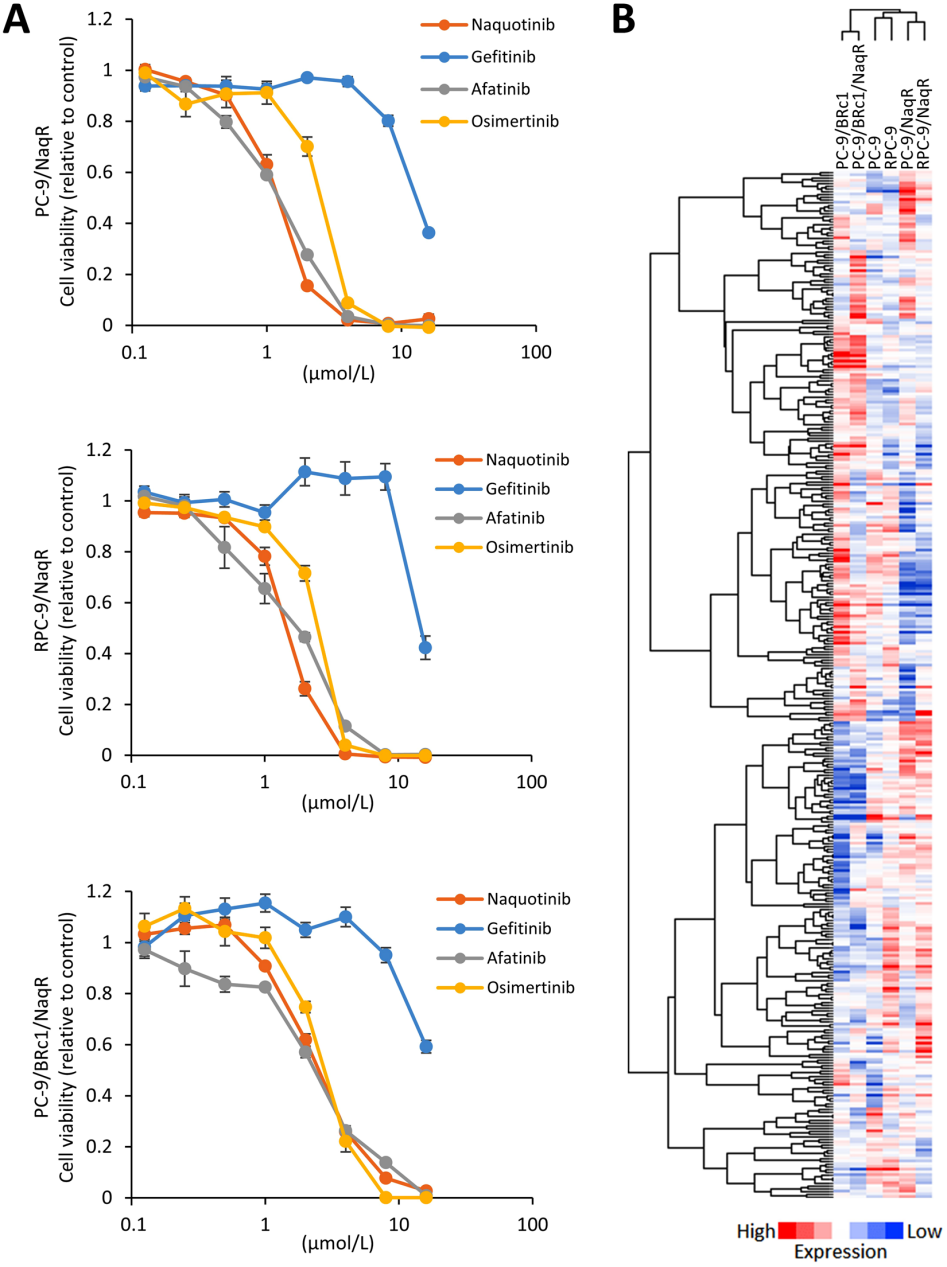
Heterogeneous gene expression of naquotinib resistance in a cell line-based model. (**A**) MTT assays following treatment with the indicated concentrations of EGFR-TKIs in PC-9/NaqR, RPC-9/NaqR, and PC-9/BRc1/NaqR cells. Error bars, standard error (SE). All experiments were performed in triplicate. (**B**) Heatmap of targeted RNA-sequencing analysis of 612 kinases and kinase-related genes from parental cells and naquotinib-resistant cells. All RNA samples were analyzed twice and averaged.

Next, we assessed the effects of several generations of EGFR-TKIs in these naquotinib-resistant cell lines. The resistant cell lines exhibited 52- to 157-fold resistance to naquotinib compared with each parental cell line *in vitro* (Table 1). These cell lines also showed cross-tolerance to other EGFR-TKIs, such as gefitinib, afatinib, and osimertinib (Fig. 1A). Direct sequencing revealed that these resistant cells preserved the original activating mutation in *EGFR* exon 19 or 20. Furthermore, the acquired *EGFR* C797S mutation, which confers resistance to osimertinib^13^, was not detected in any of the three cell lines (Supplementary Fig. 3A–C). Epithelial-to-mesenchymal transition was not detected in any of the resistant cell lines (Supplementary Fig. 2D).

**Table 1:** IC_50_ values of naquotinib on parental and naquotinib-resistant cells.

Cell line	Naquotinib IC_50_ (µmol/L) values ± SE	Relative resistance
PC-9	0.026 ± 0.005	
PC-9/NaqR	1.359 ± 0.101	52
RPC-9	0.025 ± 0.002	
RPC-9/NaqR	1.522 ± 0.062	61
PC-9/BRc1	0.016 ± 0.001	
PC-9/BRc1/NaqR	2.508 ± 0.141	157

NOTE: The anti-proliferative effects were evaluated by the MTT assay. Data are presented as the mean ± standard error (SE) from three independent experiments.

Next, to assess the gene expression profiles of the resistant cells, we comprehensively analyzed RNA expression profiles using next-generation sequencing. A total of 612 human kinases and kinase-related genes were assessed. The expression profile of each resistant cell line varied, and a common targetable alteration was not found among the resistant cells (Fig. 1B, Supplementary Fig. 9). Consequently, we assessed the mechanism of resistance in each respective cell line.

### Heterogeneous MET expression in PC-9/NaqR cells

**Figure 2:**
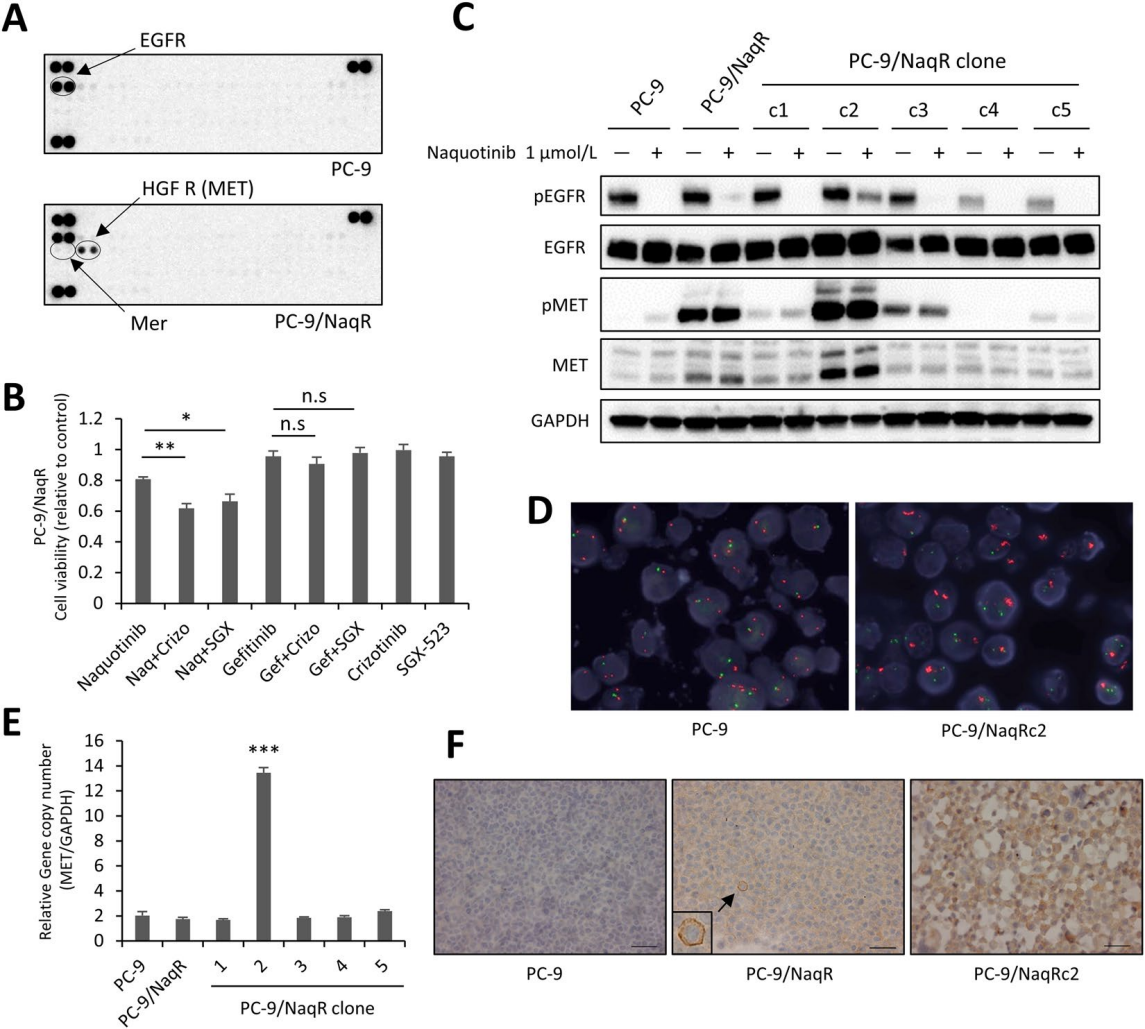
MET heterogeneity in PC-9/NaqR cells. (**A**) Phospho-receptor tyrosine kinase (RTK) arrays in PC-9 and PC-9/NaqR cells. Resistant cells were cultured in normal medium for 4 days. (**B**) Inhibitory effect of the combination therapy of EGFR-TKIs and MET inhibitors on cell proliferation in PC-9/NaqR cells (MTT assay). All drugs were exposed at 1.0 µmol/L for 96 h. Data are presented as the mean ± SE from three independent experiments. *p < 0.05; **p < 0.01; n.s., not significant. EGFR-TKIs; Gef: gefitinib, Naq: naquotinib; MET inhibitors; Crizo: crizotinib, SGX: SGX-523. (**C**) Phosphorylation of EGFR or MET signaling in PC-9/NaqR clones. Each cell line was incubated with naquotinib (0 or 1.0 µmol/L) for 4 h. (**D**) Fluorescence *in situ* hybridization (FISH) analysis of *MET* amplification in PC-9 and PC-9/NaqRc2 cells. Red, *MET* gene; green, *CEP7* gene. (**E**) Quantitative polymerase chain reaction (qPCR) analysis of the *MET* copy number. DNA was derived from PC-9, PC-9/NaqR, and PC-9/NaqR sub-clones. All experiments were performed in triplicate. Error bars, SE. ***p < 0.001. (**F**) Immunohistochemistry of MET in PC-9, PC-9/NaqR, and PC-9/NaqRc2 cells. Scale bars, 50 µm. Uncropped immunoblots are shown in Supplementary Fig. S8.

**Figure 3:**
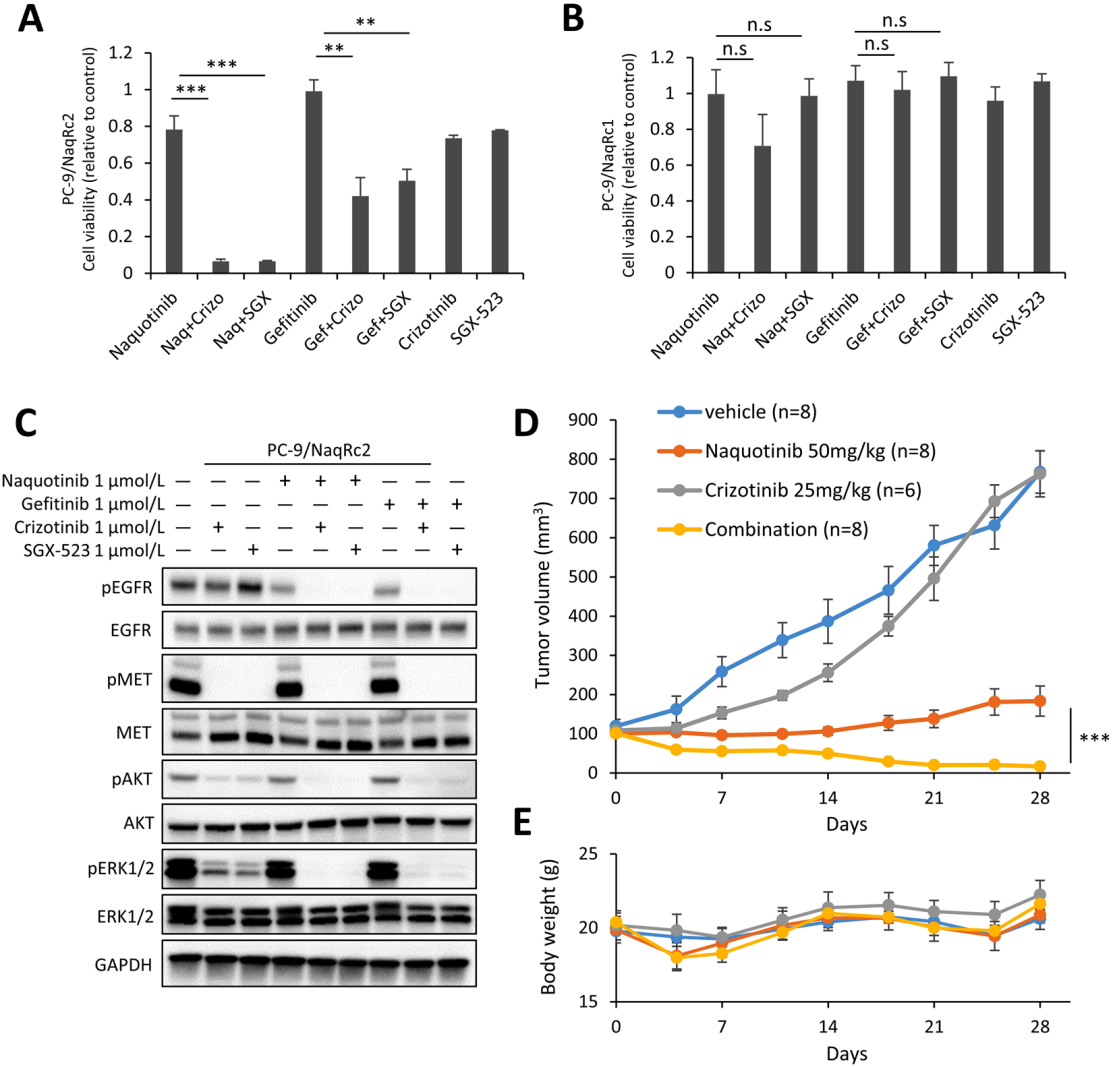
Effects of the combination therapy of EGFR-TKIs and MET inhibitors in PC-9/NaqRc2 cells. EGFR-TKIs; Gef: gefitinib, Naq: naquotinib; MET inhibitors; Crizo: crizotinib, SGX: SGX-523. (**A** and **B**) Inhibitory effect of the combination of EGFR-TKIs and MET inhibitors on cell proliferation in PC-9/NaqRc2 and PC-9/NaqRc1 cells (MTT assay). All drugs were exposed at 1.0 µmol/L for 96 h. Data are presented as the mean ± SE from three independent experiments. **p < 0.01; ***p < 0.001; n.s., not significant. (**C**) Combination effects of EGFR-TKIs and MET inhibitors on EGFR pathway signaling in PC-9/NaqRc2 cells. All drugs were exposed at 1.0 µmol/L for 4 h. (**D**) Combination effects of naquotinib and crizotinib on tumor growth in PC-9/NaqRc2 cells in xenograft models. Mice were treated with vehicle, naquotinib (50 mg/kg, five times per week p.o.), crizotinib (25 mg/kg, five times per week p.o.), or the combination for 4 weeks. ***p < 0.001. (**E**) Mouse body weight in xenograft models. Uncropped immunoblots are shown in Supplementary Fig. S8.

We first focused on PC-9/NaqR cells derived from PC-9, EGFR-TKI-naïve lung adenocarcinoma cells. The top 10 upregulated genes (relative to parental cells) are listed in Supplementary Figure 3D. The MER proto-oncogene, tyrosine kinase (MERTK), which is associated with cancer progression^21,22^, was overexpressed 9-fold in PC-9/NaqR cells relative to the parental cells. However, as MERTK inhibitors, crizotinib, sunitinib, and UNC569 did not inhibit the proliferation of the resistant cells (Fig. 2B, Supplementary Fig. 4A). We also examined the phosphorylation of MERTK using human phospho-RTK arrays. As expected, MERTK was not phosphorylated, suggesting that this protein does not play a major role in cancer proliferation or drug resistance. By contrast, MET phosphorylation was increased in PC-9/NaqR cells relative to the parental PC-9 cells (Fig. 2A). Western blot analysis confirmed the increased phosphorylation of MET (Supplementary Fig. 4B) in the resistant cells; however, the combination of the MET inhibitors crizotinib and SGX-523 with naquotinib partially inhibited cell proliferation (Fig. 2B). To explore this discrepancy, we performed single-cell cloning using a limiting dilution method. We found that one clone, designated PC-9/NaqRc2, overexpressed MET protein compared with PC-9/NaqR and other clones (e.g., PC-9/NaqRc1 and c3) (Fig. 2C). *MET* amplification was confirmed by FISH analysis in PC-9/NaqRc2 cells (Fig. 2D). Only PC-9/NaqRc2 cells showed an increased *MET* gene copy number (per *GAPDH* gene), while other clones had no copy number gain, similar to the parental cells (Fig. 2E). Immunohistochemistry revealed that MET overexpression was very minor in PC-9/NaqR cells, in contrast to PC-9/NaqRc2 cells (Fig. 2F). We hypothesized that this heterogeneity may confer resistance to MET inhibitors; therefore, we tested the effects of MET inhibitors in the resistant clone PC-9/NaqRc2. As expected, the combination of EGFR-TKIs and MET inhibitors (crizotinib or SGX-523) dramatically inhibited the proliferation of PC-9/NaqRc2 cells (Fig. 3A), but had no effect on PC-9/NaqRc1 cells (Fig. 3B). In other words, EGFR-TKI monotherapy or MET inhibitor monotherapy had little effect on cell proliferation in PC-9/NaqRc2 cells. Based on these results, such monotherapies may partially inhibit the phosphorylation of EGFR or a downstream signaling pathway, such as AKT or ERK, and the combination of EGFR-TKIs and MET inhibitors completely inhibited the phosphorylation of these signaling proteins in PC-9/NaqRc2 cells (Fig. 3C). To confirm the effects of the combination therapy, we conducted *in vivo* experiments using PC-9/NaqRc2 cells. Consistent with the *in vitro* data, the combination of naquotinib (50 mg/kg, 5 times per week) and crizotinib (25 mg/kg, 5 times per week) showed excellent inhibitory effects compared with naquotinib monotherapy (50 mg/kg, 5 times per week) or crizotinib monotherapy (25 mg/kg, 5 times per week) *in vivo*. No differences in body weight were observed (Fig. 3D and E).

To further examine the role of MET in EGFR-TKI-naïve cancer cells, we developed another resistant cell line from EGFR-TKI-naïve lung cancer cells, HCC827, which harbor the *EGFR* exon 19del. The resistant cell line, designated HCC827/NaqR, was established in the same manner as the PC-9/NaqR cell line. The human phospho-RTK array revealed activation of the MET protein, and *MET* amplification was detected by FISH and qPCR in the resistant cells (Supplementary Fig. 5A–C). In contrast to PC-9/NaqR cells, the combination of EGFR-TKIs and MET inhibitors showed an excellent inhibitory effect on cell proliferation and phosphorylation of the EGFR signaling pathway in HCC827/NaqR cells (Supplementary Fig. 5D and E). Consistent with this observation, immunohistochemistry revealed that MET overexpression was major in HCC827/NaqR cells (Supplementary Fig. 5F).

### *NRAS* amplification in RPC-9 /NaqR cells

**Figure 4:**
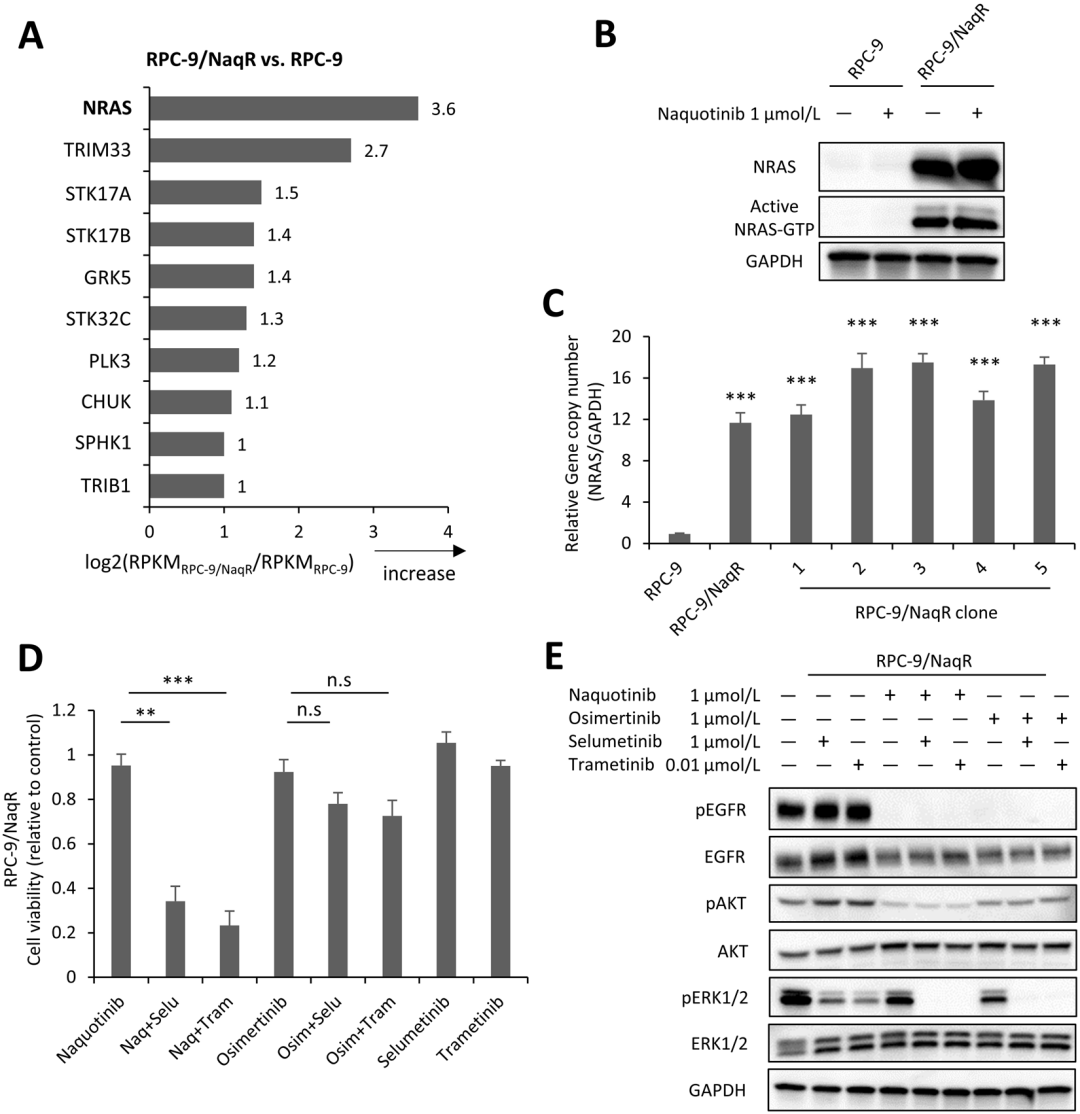
Activation of NRAS and the combination therapy of MEK inhibitors and naquotinib in RPC-9/NaqR and RPC-9/OsiR cells. EGFR-TKIs; Naq: naquotinib, Osim: osimertinib, MEK inhibitors; Selu: selumetinib, Tram: trametinib. (**A**) The gene expression profile of RPC-9/NaqR cells was compared with that of RPC-9 cells by targeted RNA-sequencing. The top 10 upregulated genes are listed. (**B**) Expression and activation of NRAS protein in RPC-9 and RPC-9/NaqR cells. Each cell line was incubated with naquotinib (0 or 1.0 µmol/L) for 4 h. (**C**) *NRAS* copy number was analyzed by qPCR. DNA was extracted from RPC-9, RPC-9/NaqR, and RPC-9/NaqR clones. Error bars, SE. ***p < 0.001. All experiments were performed in triplicate. (**D**) Inhibitory effect of MEK inhibitors and EGFR-TKIs on cell proliferation assessed in RPC-9/NaqR cells (MTT assay). All drugs were exposed at 1.0 µmol/L (except for trametinib, 0.01 µmol/L) for 96 h. Data are presented as the mean ± SE from three independent experiments. **p < 0.01; ***p < 0.001; n.s., not significant. (**E**) Effects of MEK inhibitors and EGFR-TKIs on the EGFR pathway in RPC-9/NaqR cells. All drugs were exposed at 1.0 µmol/L (except for trametinib, 0.01 µmol/L) for 4 h. Uncropped immunoblots are shown in Supplementary Fig. S8.

Next we investigated RPC-9/NaqR cells, which were derived from gefitinib-resistant lung adenocarcinoma cell lines (RPC-9 cells harboring the *EGFR* exon 19del and T790M mutations). Exposure to naquotinib inhibited the phosphorylation of EGFR and AKT in resistant and parental cells, respectively, whereas ERK1/2 phosphorylation was uniquely maintained in resistant cells (Supplementary Fig. 4C). Human phospho-RTK arrays showed no specific changes between resistant and parental cells (Supplementary Fig. 6A). Interestingly, RNA kinome sequencing analysis revealed that *NRAS* was the most overexpressed gene in RPC-9/NaqR cells compared with the parental RPC-9 cells (Fig. 4A). Other RAS-related genes, such as *KRAS* and *HRAS*, were not significantly altered (Supplementary Fig. 6B). Overexpression of NRAS protein, amplification of *NRAS*, and NRAS activation were confirmed in RPC-9/NaqR cells by Western blotting, qPCR, and NRAS-GTP pull-down assays (Fig. 4B and C). Direct sequencing of *NRAS* and real-time PCR showed no mutations in *NRAS* exon 2 or 3 (Supplementary Fig. 6C). No targetable RTK genes, including *MET* and *EGFR*, were upregulated in RPC-9/NaqR cells (Fig. 4A and Supplementary Fig. 7E). Therefore, we thought that ERK1/2 phosphorylation was maintained by the activation of NRAS, upstream of the MAPK pathway. Next, to assess the heterogeneity of this alteration, we performed single-cell cloning of PRC-9/NaqR. As a result, *NRAS* amplification was identified in all RPC-9/NaqR sub-clones, which were designated RPC-9/NaqRc1 to c5 (Fig. 4C).

### Beneficial effects of the combination therapy of MEK inhibitors and naquotinib

Next, we evaluated whether inhibition of the RAS/MAPK pathway using MEK inhibitors, such as selumetinib or trametinib, can overcome resistance to naquotinib in RPC-9/NaqR cells. Monotherapies with MEK inhibitors (selumetinib or trametinib) had little effect on the proliferation of RPC-9/NaqR cells, but the combination of MEK inhibitors and naquotinib clearly inhibited cell proliferation (Fig. 4D). Consistent with this result, monotherapies with selumetinib or trametinib partially inhibited the phosphorylation of ERK and had no effect on the phosphorylation of EGFR or AKT in PRC-9/NaqR cells, while the combination showed a great inhibitory effect on the phosphorylation of EGFR, AKT, and ERK (Fig. 4E). We next assessed the effects of the combination therapies on xenograft mouse tumors, but RPC-9/NaqR cells were unable to grow *in vivo* for unknown reasons.

To confirm the effects of the combination therapy, we tested the effects of another combination therapy (MEK inhibitors and osimertinib) in RPC-9/NaqR cells. Surprisingly, the combination therapy showed a limited effect on the proliferation of RPC-9/NaqR cells (Fig. 4D). Therefore, we assessed the difference in the inhibitory effects between the two combination therapies (MEK inhibitors with naquotinib or osimertinib). Western blot analysis revealed no difference in the phosphorylation of EGFR or ERK between the two therapies. However, AKT phosphorylation was relatively reduced in cells treated with both MEK inhibitors and naquotinib or naquotinib monotherapy compared with cells treated with MEK inhibitors and osimertinib or osimertinib monotherapy (Fig. 4E). To further explore this difference, we screened phosphorylated proteins involved in the AKT pathway using a PathScan AKT Pathway Array Kit (Cell Signaling Technology) (Supplementary Fig. 6D). The array confirmed the greater effect of naquotinib on the phosphorylation of AKT when compared with osimertinib (Supplementary Figs 6E and 10). Naquotinib inhibited the phosphorylation of most AKT-related proteins when compared to osimertinib (Supplementary Figs 6E and 10). Additionally, naquotinib plus selumetinib inhibited the phosphorylation of all AKT-related proteins compared to osimertinib plus selumetinib. Naquotinib plus selumetinib showed a greater effect, especially on the phosphorylation of AKT-related proteins such as PRAS40, GSK-3beta, PDK1 or 4E-BP1 (Supplementary Figs 6E and 10). Furthermore, we re-examined the RNA kinome sequencing data, but no genes related to the AKT pathway were up- or downregulated in RPC-9/NaqR cells (Supplementary Fig. 6B).

**Figure 5:**
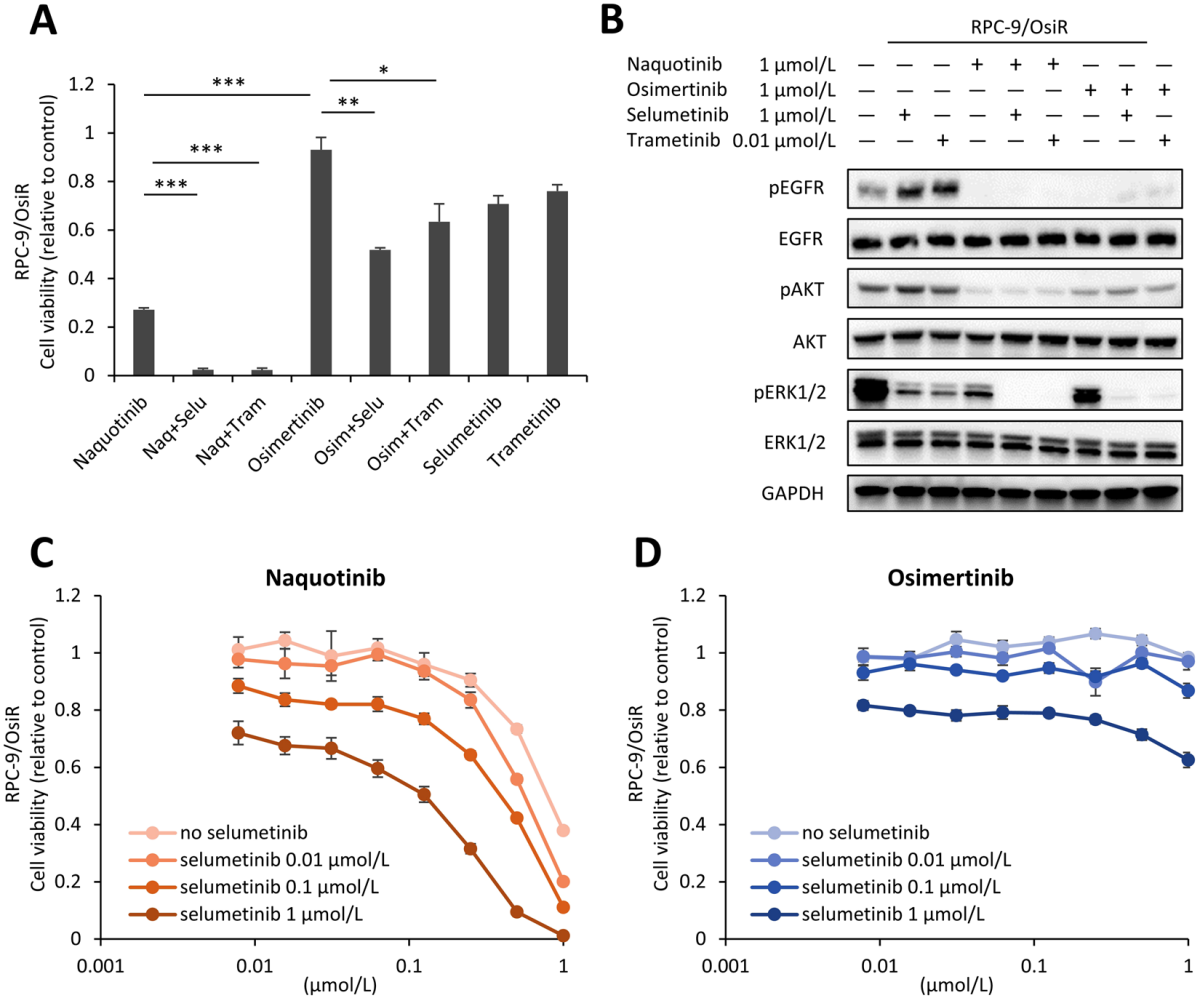
Effects of the combination therapy of MEK inhibitors and naquotinib in RPC-9/OsiR cells. EGFR-TKIs; Naq: naquotinib, Osim: osimertinib, MEK inhibitors; Selu: selumetinib, Tram: trametinib. (**A**) Inhibitory effect of MEK inhibitors and EGFR-TKIs on cell proliferation assessed in RPC-9/OsiR cells (MTT assay). All drugs were exposed at 1.0 µmol/L (except for trametinib, 0.01 µmol/L) for 96 h. Data are presented as the mean ± SE from three independent experiments. *p < 0.05; **p < 0.01; ***p < 0.001. Naq, naquotinib; Selu, selumetinib; Tram, trametinib; Osim, osimertinib. (**B**) Combination effects of MEK inhibitors and EGFR-TKIs on EGFR pathway signaling in RPC-9/OsiR cells. All drugs were exposed at 1.0 µmol/L (except for trametinib, 0.01 µmol/L) for 4 h. (**C**) Inhibitory effect of naquotinib and selumetinib (0.01 to 1 µmol/L) in RPC-9/OsiR cells. (**D**) Inhibitory effect of osimertinib and selumetinib (0.01 to 1 µmol/L) in RPC-9/OsiR cells. C.D. Error bars, SE. All experiments were performed in triplicate. Uncropped immunoblots are shown in Supplementary Fig. S8.

Finally, we tested the effects of the combination therapies on cell proliferation in osimertinib-resistant cell lines. Similar to RPC-9/NaqR cells, the osimertinib-resistant cell lines were derived from RPC-9 cells, designated RPC-9/OsiR (Supplementary Fig. 7A). RPC-9/OsiR cells maintained the *EGFR* T790M mutation and had no *EGFR* C797S mutation (Supplementary Fig. 7B). The human phospho-RTK array revealed no specific changes (Supplementary Fig. 7C). No *MET* amplification was detected in RPC-9/OsiR cells (Supplementary Fig. 7E). NRAS overexpression and activation were observed in RPC-9/OsiR cells (Supplementary Fig. 7D). Osimertinib monotherapy or the combination therapy of MEK inhibitors and osimertinib showed a limited inhibitory effect on cell proliferation in RPC-9/OsiR cells. By contrast, the combination of MEK inhibitors and naquotinib dramatically inhibited cell proliferation in RPC-9/OsiR cells (Fig. 5A). Similar to RPC-9/NaqR cells, the combination therapy of MEK inhibitors and naquotinib showed a greater inhibitory effect on the phosphorylation of AKT than the combination of MEK inhibitors and osimertinib in RPC-9/OsiR cells (Fig. 5B). An additive effect of the MEK inhibitor selumetinib was consistently observed with naquotinib in a dose-dependent manner, but no effect was observed with osimertinib in RPC-9/OsiR cells (Fig. 5C and D).

## Discussion

Elucidation of the mechanism of resistance to 3^rd^-gen EGFR-TKIs and the development of a novel strategy for drug resistance are priorities in *EGFR*-mutant NSCLC. In this study, we discovered a highly beneficial effect from the combination therapy of MEK inhibitors and naquotinib. This combination therapy inhibited cell proliferation in both naquotinib-resistant and osimertinib-resistant cells, although the combination of MEK inhibitors and osimertinib had a limited effect on these resistant cancer cells. The precise mechanisms remain to be clarified to explain why the combination of MEK inhibitors and naquotinib had a greater effect. One possible explanation is an off-target inhibitory effect of naquotinib on the AKT pathway. Another possible mechanism is the synergistic effect of naquotinib and MEK inhibitors on the AKT pathway (e.g., PDK1; Supplementary Figs 6E and 10).

We also determined that *MET* or *NRAS* amplification may represent a clinically targetable mechanism of resistance to naquotinib. Several studies have reported that *MET* amplification conferred resistance to EGFR-TKIs, including 3^rd^-gen EGFR-TKIs^16,17,23–25^. Our study shows that treatment with naquotinib also induced *in vitro MET* amplification in lung cancers with *EGFR* mutations. Similar to other preclinical studies^23–28^, combination therapy with naquotinib and MET inhibitors exerted promising effects in lung tumors with *EGFR* mutations and *MET* amplification (Fig. 3). The effects of combined therapy with EGFR-TKIs and MET-TKI have been reported in clinical studies^29,30^. Considering the clinical results and inhibitory profiles, the combination of osimertinib plus savolitinib or gefitinib plus savolitinib may be the most promising therapy in lung tumors with *EGFR* mutations and *MET* amplification. *NRAS* amplification also conferred resistance to naquotinib in RPC-9/NaqR cells. Other studies have shown that activation of the RAS pathway is a resistance mechanism of EGFR-TKIs^5,14^. MEK inhibitors are the most promising agent for lung cancer with RAS activation, although RAS-specific inhibitors are not clinically available^31,32^. Combination therapies with EGFR-TKIs and MEK inhibitor have been developed for lung cancers characterized by *EGFR* mutations (NCT02143466 and NCT02025114). Consequently, the combination of 3^rd^-gen EGFR TKIs and MEK inhibitors may be a reasonable strategy for the treatment of lung cancers with *EGFR* mutations and RAS activation.

Currently, osimertinib is the standard care for patients with lung tumors harboring *EGFR* T790M; however, acquired resistance is inevitable. Therefore, a next-generation treatment strategy is warranted in the osimertinib era. Multiple drug-resistant mechanisms of each 3^rd^-gen EGFR-TKI have already been elucidated in clinical samples. For example, the mechanism of resistance to osimertinib has been described as follows: acquired resistant mutation of *EGFR* C797S^13^, *MET* amplification^17^, *FGFR1* amplification^33^, EGFR ligand overexpression^33^, and acquired* BRAF* V600E mutation^34^. The mechanism of resistance to another 3^rd^-gen EGFR-TKI, rociletinib, has been described as follows: novel resistant mutation of L798I^25^, bypass signaling of *MET* or *HER2* amplification^25^, and MAPK pathway activation by PIK3CA or KRAS^25^. In this preclinical study, the mechanisms of resistance to naquotinib were as follows: *MET* amplification in PC-9 or HCC827 cells treated as a first line treatment, and *NRAS* amplification in RPC-9 cells treated as a second line treatment. Consequently, the pattern of resistance mechanism to each 3^rd^-gen EGFR-TKI may differ, which could be due to different inhibitory profiles and treatment sequence. Additionally, osimertinib exhibited a certain effect in clinical lung cancers treated with other 3^rd^-gen EGFR-TKIs, such as rociletinib and nazartinib^35,36^. Considering these results, another type of 3^rd^-gen EGFR-TKI, such as naquotinib, may be able to inhibit lung tumors that have acquired resistance to osimertinib. Moreover, based on our preclinical data (Fig. 5), the combination therapy of MEK inhibitors and naquotinib may have a better ability to inhibit osimertinib-resistant tumors in the clinic.

It is necessary to discuss the optimal sequence of EGFR-TKIs that prolongs patient survival. In our pre-clinical model, multiple gene alterations were detected in naquotinib-resistant cells. Some of these alterations (e.g., *MET* or *NRAS* amplification) are clinically targetable, but the others appear to be un-targetable. PC-9/BRc1/NaqR cells showed multiple gene alterations compared to the parental PC-9/BRc1 cells; however, we did not identify any clinically targetable genes among them. Although the FLAURA trial (NCT02296125) showed that osimertinib resulted in better progression-free survival than first-generation EGFR-TKIs in EGFR-TKI-naïve patients, we should be careful when using 3^rd^-gen EGFR-TKIs for first-line treatment until confirmed survival results are available. Preclinical studies, including ours, have revealed that lung cancer cells easily acquire resistance to 3^rd^-gen EGFR-TKIs *in vitro*, and some resistant cells could be clinically un-targetable, which may narrow the long-term clinical benefits of EGFR-TKIs. In fact, the SOLAR trial (NCT02588261), which evaluated the use of naquotinib in EGFR-TKI-naïve patients, was terminated early for unknown reasons.

Accumulating evidence has revealed that tumor heterogeneity plays an important role in drug resistance^37–39^. In our model, a trunk mutation (oncogenic *EGFR* mutation) was detected in all resistant cell lines; however, the branch alterations varied. Only one of the single-cell clones derived from PC-9/NaqR cells harbored an *MET* amplification. As a result, MET inhibitors exhibited an inhibitory effect in PC-9/NaqRc2 cells but did not affect the other PC-9/NaqR cells or other clones without *MET* amplification. These results suggest the difficulty of managing heterogeneous tumors. Tumor evolution may be complicated further with multiple treatments^39^. Therefore, intensive combination treatment early with another class of agents, such as an anti-angiogenic antibody, an anti-EGFR antibody, or cytotoxic chemotherapy, may represent an alternative strategy for treatment of naïve patients^40,41^.

In conclusion, combination therapy with MEK inhibitors and naquotinib exerted promising effects on osimertinib-resistant lung cancer cells in a pre-clinical model. Clinical development of naquotinib monotherapy was discontinued, but the clinical assessment of this novel combination is worth considering in osimertinib-resistant lung tumors in the osimertinib era.

## Electronic supplementary material

Supplementary information
