# Supplementary material for: MET or NRAS amplification is an acquired resistance mechanism to the third-generation EGFR inhibitor naquotinib
Source: Sci Rep. 2018 Jan 31;8:1955. doi: 10.1038/s41598-018-20326-z (PMC5792548; doi:10.1038/s41598-018-20326-z)
Supplement: Supplementary file 1 — Supplementary information [file 41598_2018_20326_MOESM1_ESM.pdf]

Supplementary information

Title

MET or *NRAS* amplification is an acquired resistance mechanism to the third-generation EGFR inhibitor naquotinib

Authors

Kiichiro Ninomiya1, Kadoaki Ohashi1, 2*, Go Makimoto1, Shuta Tomida3, Hisao Higo1, Hiroe Kayatani1, Takashi Ninomiya1, Toshio Kubo4, Eiki Ichihara2, Katsuyuki Hotta5, Masahiro Tabata4, Yoshinobu Maeda1, Katsuyuki Kiura2

Author's affiliation

1. Department of Hematology, Oncology and Respiratory Medicine, Okayama University Graduate School of Medicine, Dentistry and Pharmaceutical Sciences,

2. Department of Respiratory Medicine, Okayama University Hospital,

3. Department of Biobank, Okayama University Graduate School of Medicine, Dentistry and Pharmaceutical Sciences,

4. Center for Clinical Oncology, Okayama University Hospital,

5. Center for Innovative Clinical Medicine, Okayama University Hospital

*Corresponding should be addressed to:

Kadoaki Ohashi, M.D., Ph.D.

Department of Respiratory Medicine

Okayama University Hospital

2-5-1, Shikata-cho, Okayama, 700-8558, Japan

PHONE: +81-86-235-7227

FAX: +81-86-232-8226

E-mail: kohashi@cc.okayama-u.ac.jp

Short title: Acquired resistance mechanism to the third-generation EGFR inhibitor naquotinib

Key words: EGFR T790M, Naquotinib

Supplementary Materials and Methods

Short Tandem Repeat Polymerase Chain Reaction Analysis

The genetic identity between each resistant cell line and parental cell line was confirmed by short tandem repeat polymerase chain reaction analysis using the AuthentiFiler PCR Amplification Kit (Applied Biosystems) according to the manufacturer's instructions.

Akt Signaling Array

A PathScan Akt Signaling Antibody Array Kit was purchased from Cell Signaling Technology and used according to the manufacturer's recommendations. Bands and dots were detected on an ImageQuant LAS-4000 imager.

Supplementary Figure Legends

Supplementary Figure S1.

Inhibitory effect of naquotinib on cell proliferation and the EGFR signaling pathway in PC-9 and RPC-9 cells.

A. Cell proliferation assay in PC-9, RPC-9, and PC-9/BRc1 cells treated with naquotinib or gefitinib. Error bars, SE. All experiments were performed in triplicate. B. Effects of naquotinib or gefitinib on EGFR pathway signaling in PC-9, RPC-9, and PC-9/BRc1 cells. Each cell line was incubated with naquotinib or gefitinib (0 to 1.0 μmol/L) for 4 h.

Supplementary Figure S2.

Establishment of naquotinib-resistant cell lines.

A. Schematic. Parental cells were cultured with increasing concentrations of naquotinib (beginning at half of the IC_{50}) to establish naquotinib-resistant cell lines. The dose was increased to 1.0 μmol/L in a stepwise pattern. Fresh drug was added every 96 h. Clones were derived from PC-9/NaqR and RPC-9/NaqR cells using a single-cell cloning method. B. Time course of the development of naquotinib-resistant cell lines. C. Identical test by short tandem repeats analysis between parental and resistant cells. D. Feature of the epithelial-mesenchymal transition in each naquotinib-resistant cell line. All cells were treated with 1.0 μmol/L naquotinib for 4 h. 293T cell lysate was used as an indicator of the epithelial-mesenchymal transition.

Supplementary Figure S3.

A to C. Direct sequencing of EGFR exons 19 and 20 in naquotinib-resistant cell lines. A. PC-9 and PC-9/NaqR cells. B. RPC-9 and RPC-9/NaqR cells. C. PC-9/BRc1 and PC-9/BRc1/NaqR cells. D and E. Targeted RNA-sequencing analysis of 612 kinases and kinase-related genes from parental and naquotinib-resistant cell lines. D. Changes in the RPKM levels of PC-9/NaqR cells against PC-9 cells. The top 10 upregulated genes relative to parental cells are listed. E. Changes in the RPKM levels of PC-9/BRc1/NaqR cells against PC-9/BRc1 cells. The top 10 upregulated genes relative to parental cells are listed.

Supplementary Figure S4.

Effects of MET inhibitors in PC-9/NaqR cells. EGFR-TKIs: Naq; naquotinib, Gef; gefitinib, MET inhibitors: Suni; sunitinib, UNC; UNC563.

A. Co-inhibitory effect of MET inhibitors and EGFR-TKIs in PC-9/NaqR cells. Inhibitory effect of EGFR-TKIs and MET inhibitors on cell proliferation in PC-9/NaqR cells. All drugs were exposed at 1.0 μmol/L for 96 h.

B. Effect of naquotinib on the EGFR signaling pathway in PC-9 and PC-9/NaqR cells. Each cell line was incubated with naquotinib (0 to 1.0 µmol/L) for 4 h. C. Effect of naquotinib on the EGFR signaling pathway in RPC-9 and RPC-9/NaqR cells. Each cell line was incubated with naquotinib (0 to 1.0 µmol/L) for 4 h.

Supplementary Figure S5.

Establishment of naquotinib-resistant cell lines (HCC827/NaqR from HCC827 cells) harboring the EGFR exon 19 deletion. EGFR-TKIs: Gef; gefitinib, Naq; naquotinib, MET inhibitors: Crizo; crizotinib, SGX; SGX-523.

A. Phospho-RTK arrays in HCC827 and HCC827/NaqR cells. Resistant cells were cultured in the absence of naquotinib for 5 days. B. FISH analysis of the MET gene in HCC827 and HCC827/NaqR cells. Red, MET gene; green, CEP7 gene. C. MET copy number. DNA derived from HCC827 and HCC827/NaqR cells. Each copy number relative to GAPDH was determined by qPCR. Error bars, SE. ***, $p < 0.001$. All experiments were performed in triplicate. D. Inhibitory effect of EGFR-TKIs and MET inhibitors on cell proliferation in HCC827/NaqR cells. All drugs were exposed at 1.0 µmol/L for 96 h. Data are presented as the mean ± SE from three independent experiments. **, $p < 0.01$; ***, $p < 0.001$. E. Effects of EGFR-TKIs and MET inhibitors on the EGFR pathway in HCC827/NaqR cells. All drugs were exposed at 1.0 µmol/L for 4 h. F. Immunohistochemistry of MET in HCC827 and HCC827/NaqR cells. Scale bars, 50 µm.

Supplementary Figure S6.

Effects of the combination of MEK inhibitors and naquotinib in RPC-9/NaqR cells. EGFR-TKIs; Osim: osimertinib, Naq: naquotinib, MEK inhibitors; Selu: selumetinib.

A. Phospho-RTK arrays in RPC-9 and RPC-9/NaqR cells. Resistant cells were cultured in normal medium in the absence of naquotinib for 4 days. B. Changes in the RPKM levels of RPC-9/NaqR cells relative to RPC-9 cells by RNA-sequencing transcriptome analysis for 612 kinases and kinase-related genes. Key genes related to the PI3K/AKT signaling are shown. C. Direct sequencing of NRAS exons 2 and 3 in RPC-9 and RPC-9/NaqR cells. D and E. Effects of the combination of EGFR-TKIs and selumetinib in RPC-9/NaqR cells using the PathScan AKT Pathway Array Kit. All drugs were exposed at 1.0 µmol/L for 4 h. Expression levels shown in the bar graph were derived from the average of two dots from the PathScan AKT Pathway Array Kit.

Supplementary Figure S7.

Establishing osimertinib-resistant cell lines (RPC-9/OsiR derived from RPC-9 cells) harboring the EGFR exon 19 deletion and the exon 20 T790M mutation.

A. Cellular proliferation of RPC-9/OsiR cells treated with the indicated concentrations of EGFR-TKIs. Error bars, SE. All experiments were performed in triplicate. B. Direct sequencing analysis of EGFR exon 20 in RPC-9/OsiR cells. C. Phospho-RTK arrays in RPC-9 and RPC-9/OsiR cells. Resistant cells were cultured in normal medium for 4 days. D. Effects of osimertinib on NRAS expression and activation in RPC-9 and RPC-9/OsiR cells. Each cell line was incubated with osimertinib (0 or 1.0 μmol/L) for 4 h. Cell lysates were analyzed by Western blotting. E. Quantitative polymerase chain reaction (qPCR) analysis of the *MET* copy number. The DNA samples were derived from RPC-9, RPC-9/NaqR, RPC-9/OsiR, PC-9, and PC-9/NaqRc2 cells. All experiments were performed in triplicate. Error bars, SE.

Supplementary Figure S8.
Uncropped immunoblot images for Figs. 2A, 2C, 3C, 4B, 4E, and 5B.

Supplementary Figure S9.
Changes in the RPKM levels of naquotinib-resistant cells against parental cells. The top 10 upregulated genes or downregulated genes relative to parental cells are listed.

Supplementary Figure S10.
Effects of the combination of EGFR-TKIs and selumetinib in RPC-9/NaqR cells using the PathScan AKT Pathway Array Kit. All drugs were exposed at 1.0 μmol/L for 4 h. Expression levels shown in the bar graph were derived from the average of two dots from the PathScan AKT Pathway Array Kit. EGFR-TKIs; Osim: osimertinib, Naq: naquotinib, MEK inhibitors; Selu: selumetinib.

Supplementary Table S1. Primer and probe sequences

**(A) Quantitative PCR analysis**		
Gene		Sequence
**Human MET**	Forward	5’-CCAATTTCTGACCGAGGGAATC-3’
	Reverse	5’-TCCATGTTTCATGTATGGTAGGAC-3’
	Probe	5’-CGGAGACCCTTCACTTCGCAGGCA-TAMRA-3’
**Human NRAS**	Forward	5’-CAGTGCCATGAGAGACCAATACA-3’
	Reverse	5’-CATCCTTTCAGAGAAAATAATGCTCC-3’
	Probe	5’-CAGGCGAAGGCTTCCTCTGTGTATTTGCC-TAMRA-3’
**Human GAPDH**	Forward	5’-GGCTCCCACCTTTCTCATCC-3’
	Reverse	5’-GCAGCGTACTCCCCACATC-3’
	Probe	5’-CCCCTCTACCTCCCTCCCCACCTTGA-TAMRA-3’

**(B) Direct sequencing analysis**		
Gene		Sequence
**Human EGFR exon 19**	Forward	5’-GCAATATCAGCCTTAGGTGCGGCTC-3’
	Reverse	5’-CATAGAAAGTGAACATTTAGGATGTG-3’
**Human EGFR exon 20**	Forward	5’-ACTGACGTGCCTCTCCCTCC-3’
	Reverse	5’-CCGTATCTCCCTTCCCTGATT-3’
**Human NRAS exon 2**	Forward	5’-ACCAAATGGAAGGTCACACTAGGGTTT-3’
	Reverse	5’-ACAGGATCAGGTCAGCGGGC-3’
**Human NRAS exon 3**	Forward	5’-TGAGGGACAAACCAGATAGGCAGA-3’
	Reverse	5’-CCCTAGTGTGGTAACCTCATTTCCCCA-3’

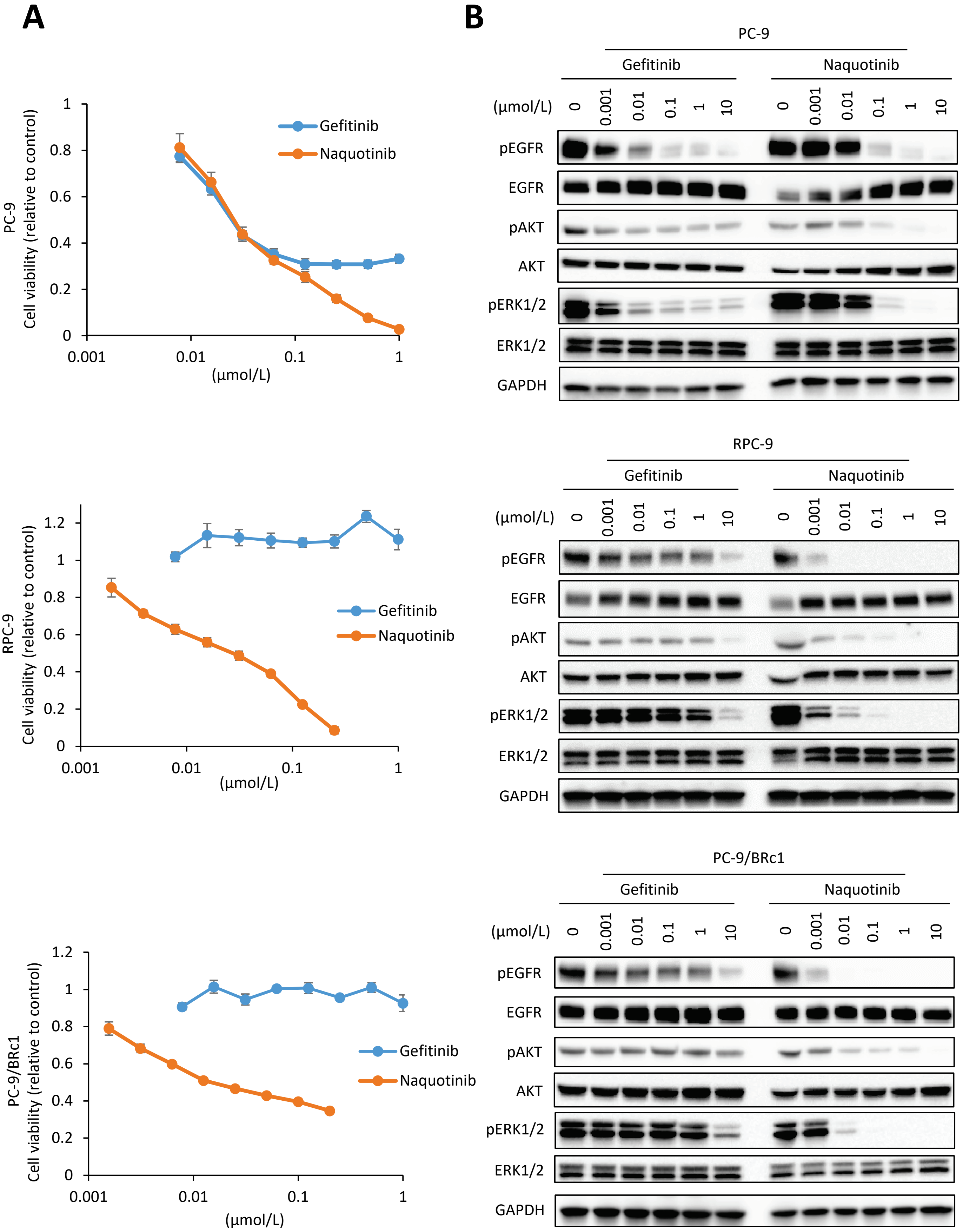

Supplementary Figure S1.

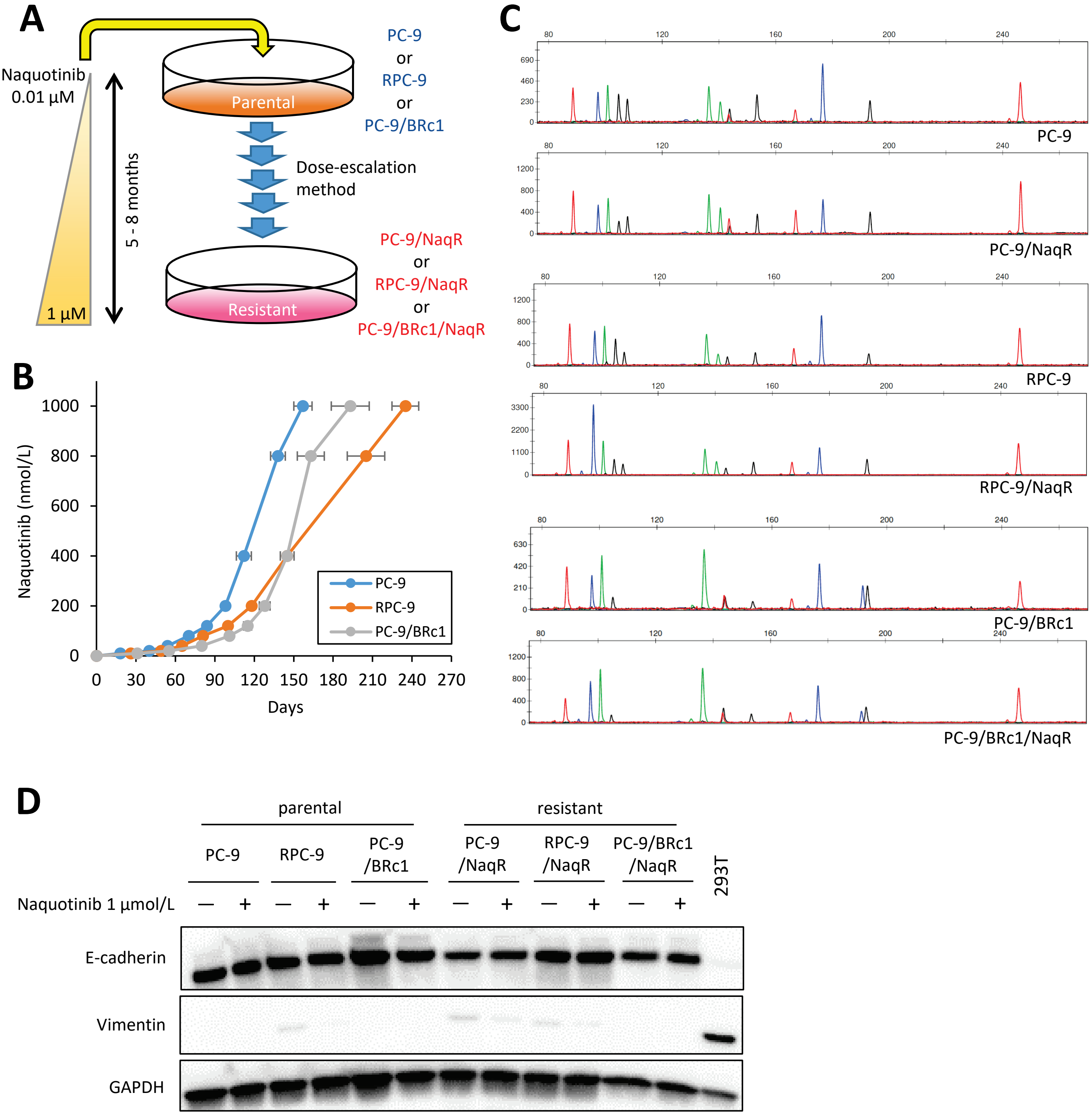

Supplementary Figure S2.

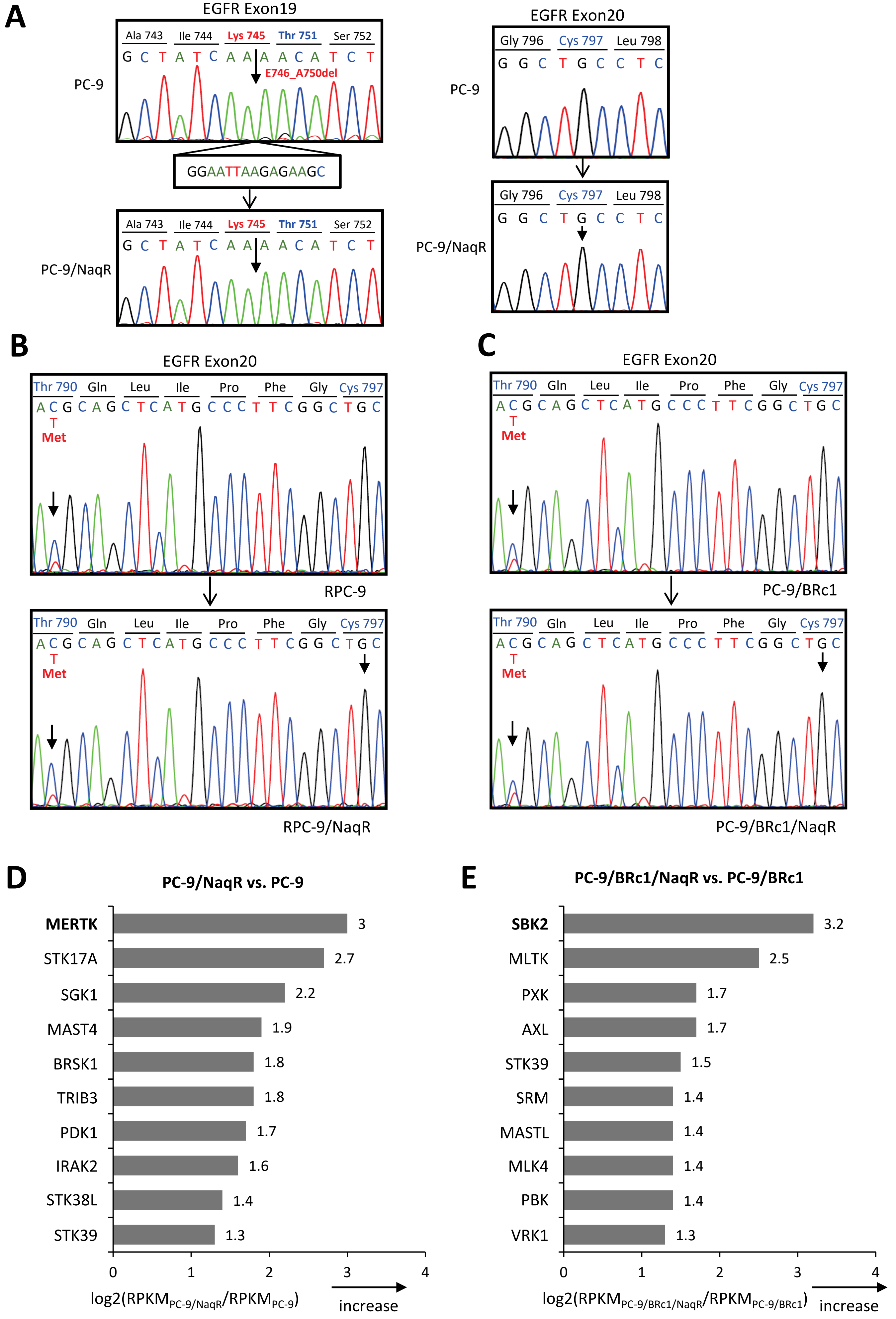

Supplementary Figure S3.

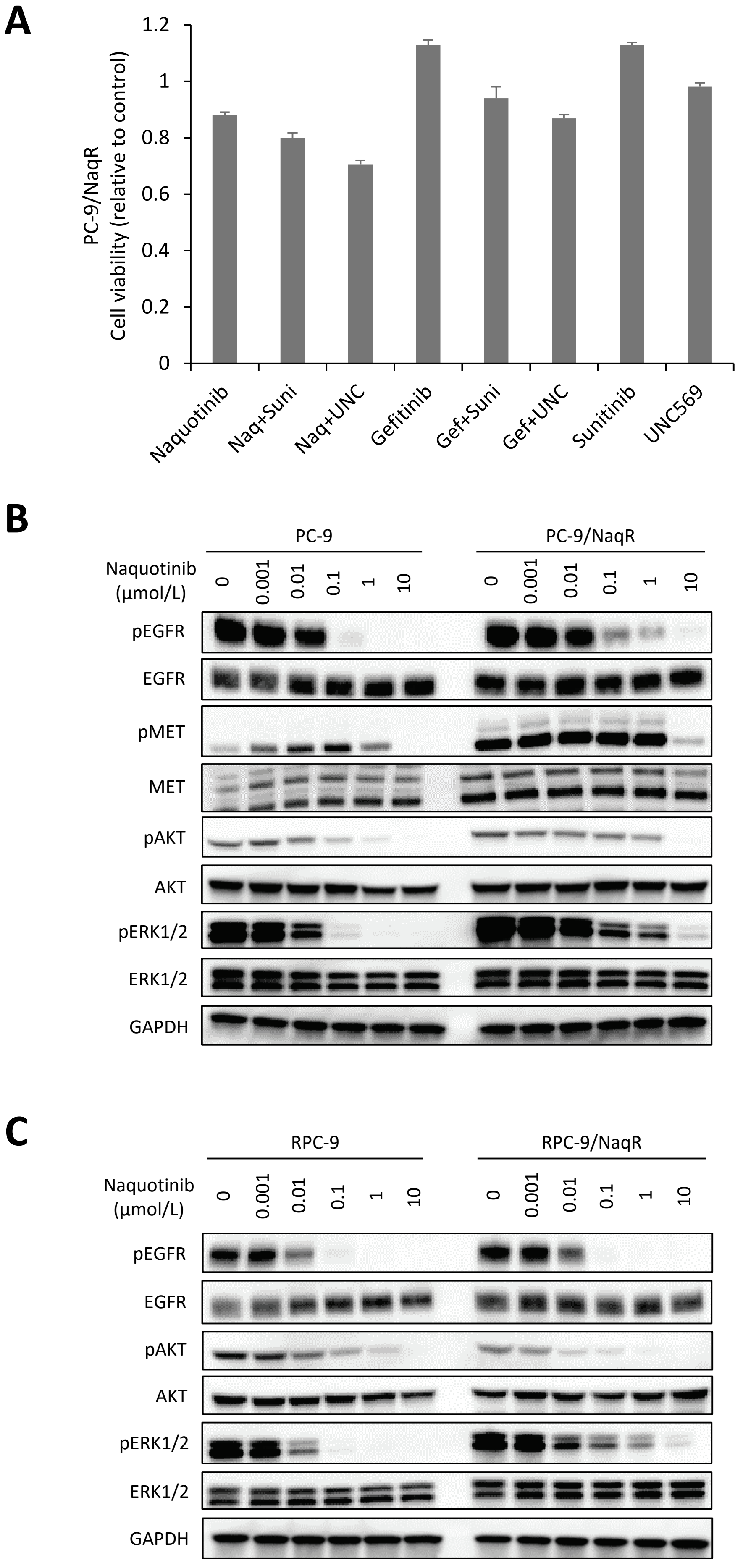

Supplementary Figure S4.

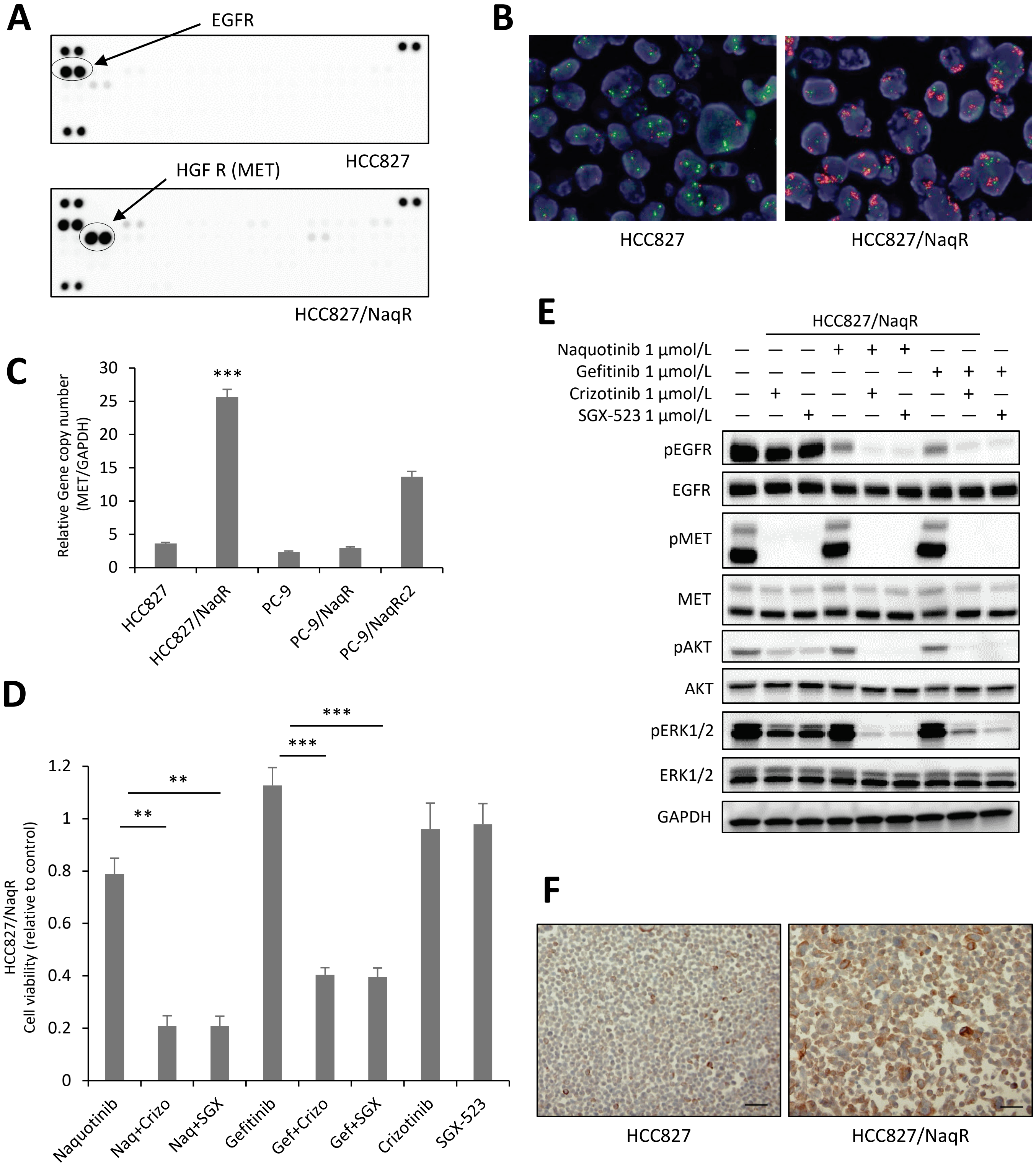

Supplementary Figure S5.

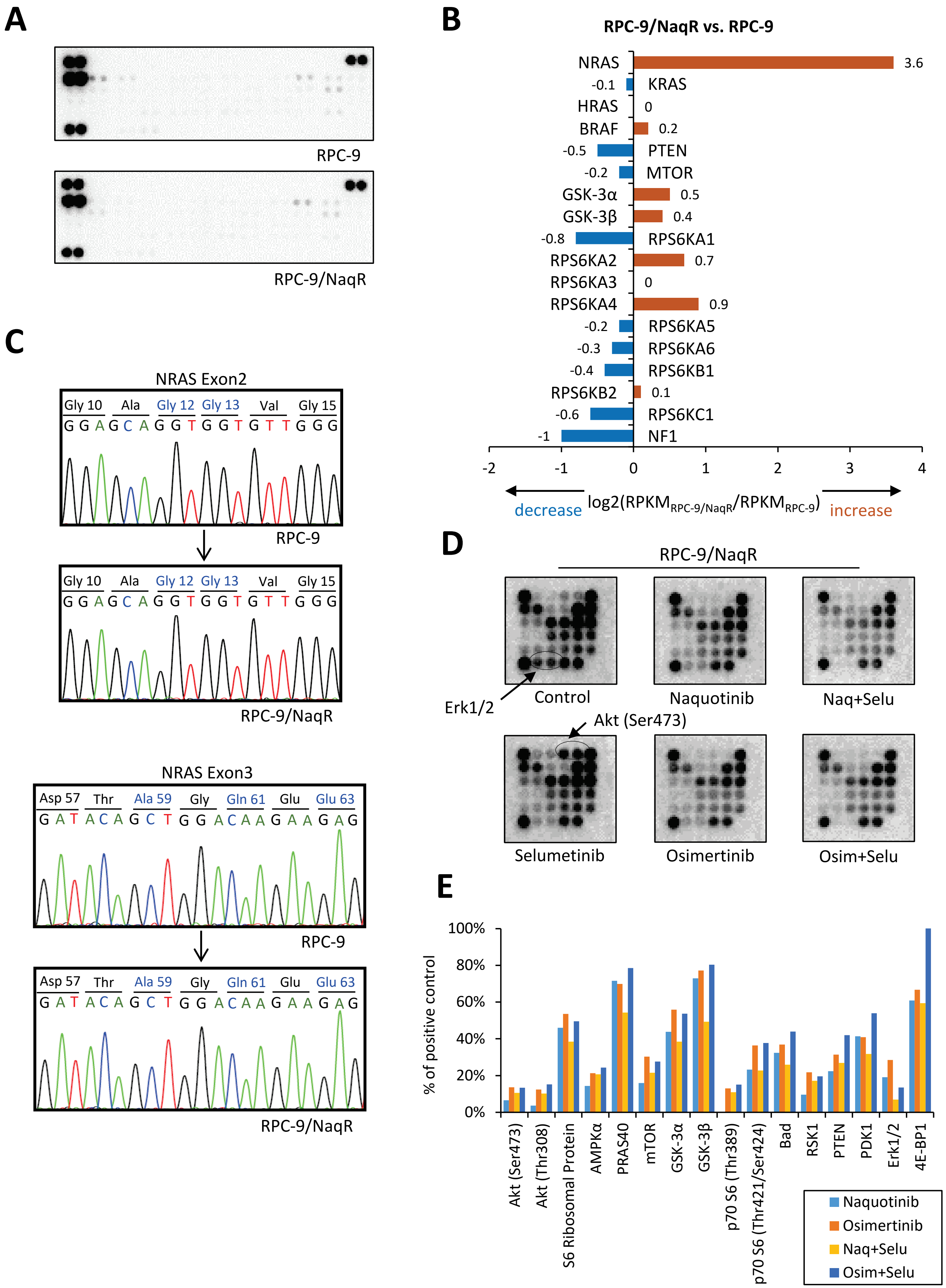

Supplementary Figure S6.

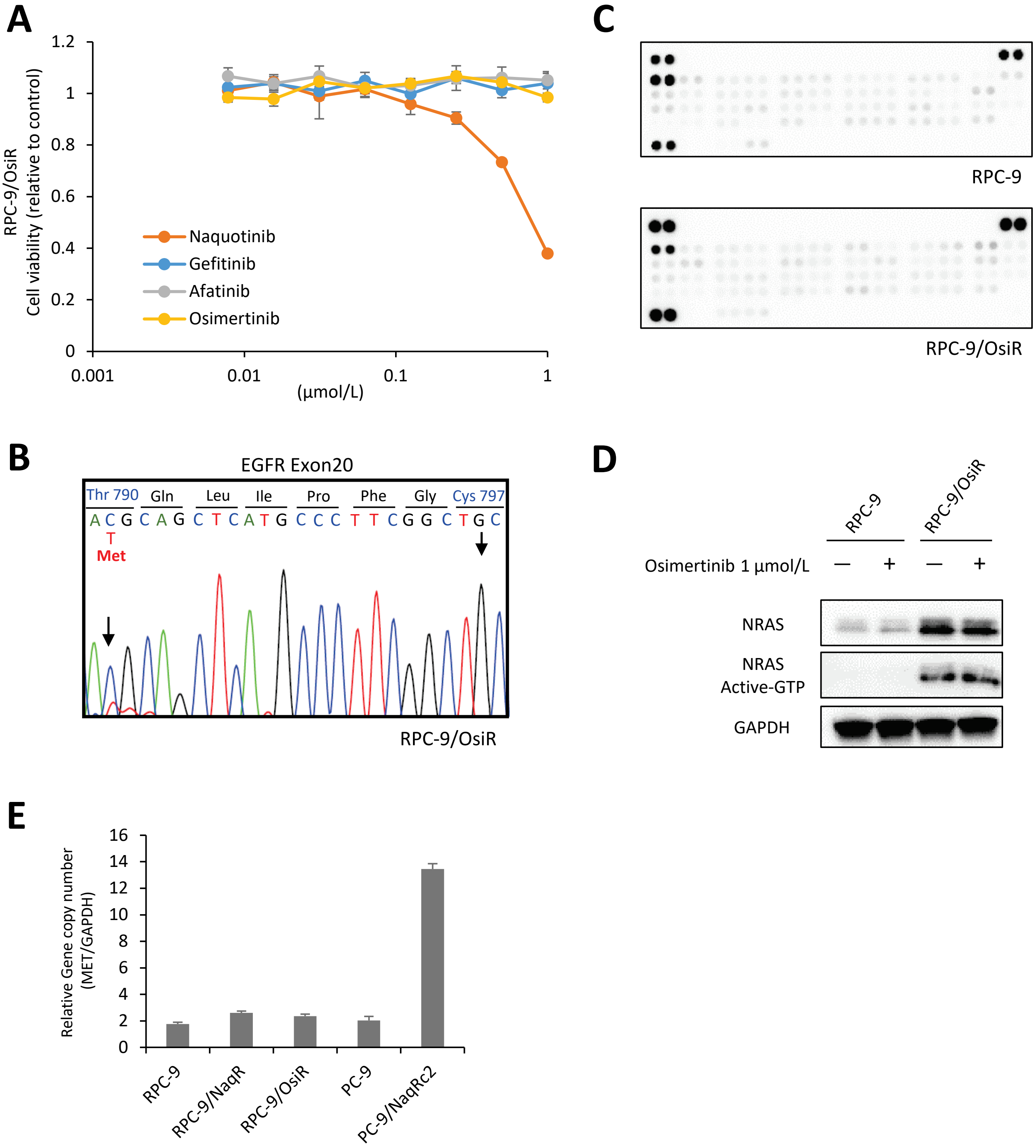

Supplementary Figure S7.

Figure 2A.

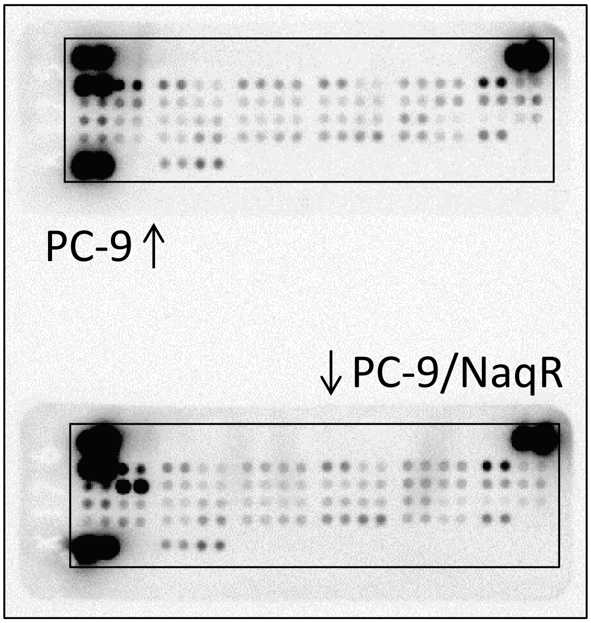

Figure 2C.

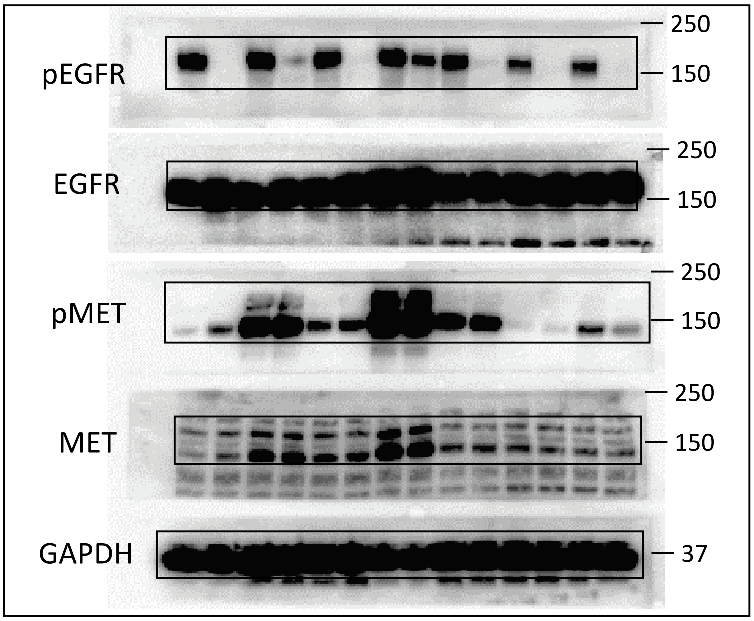

Figure 3C.

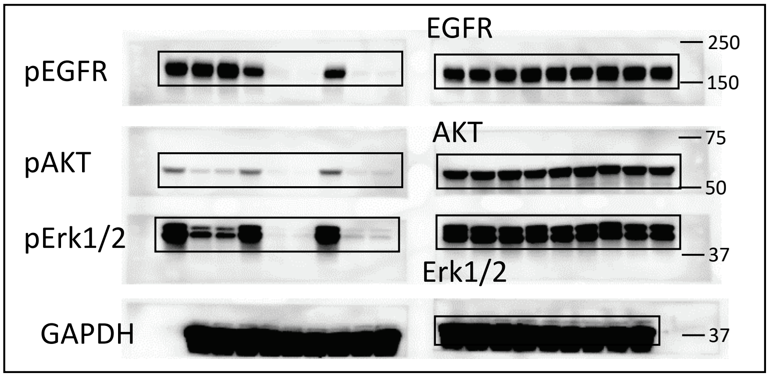

Figure 4B.

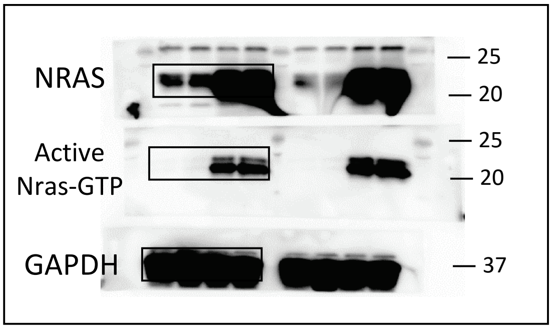

Figure 4E.

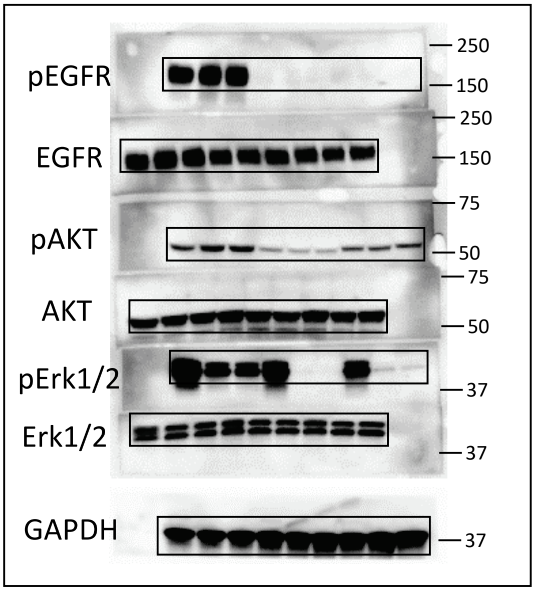

Figure 5B.

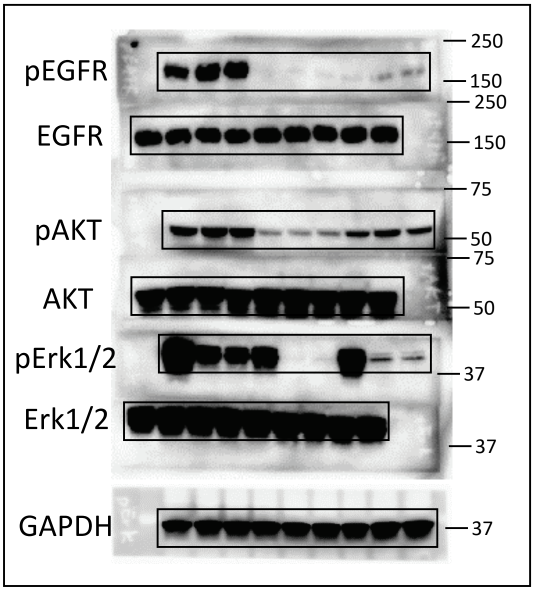

Supplementary Figure S8.

Uncropped immunoblot images for indicated figures.

PC-9/NaqR vs. PC-9	RPC-9/NaqR vs. RPC-9	PC-9/BRc1/NaqR vs. PC-9/BRc1
MERTK	NRAS	SBK2
STK17A	TRIM33	MLTK
SGK1	STK17A	PXK
MAST4	STK17B	AXL
BRSK1	GRK5	STK39
TRIB3	STK32C	SRM
PDK1	PLK3	MASTL
IRAK2	CHUK	MLK4
STK38L	SPHK1	PBK
STK39	TRIB1	VRK1

High Expression

DAPK1	MAP3K14	EPHA4
EPHB6	EPHB6	PTK6
GATA3	BMPR2	FGFR2
CDK14	DMPK	IRS2
ERBB3	PRKCZ	LIMK2
SBK1	RIPK4	STK36
DMPK	ERBB2	RIPK4
ROR1	TP53	FGFR3
STK38	PIK3C2B	ULK1
PIK3C2B	PRKCA	CDKN2B

Low Expression

Supplementary Figure S9.

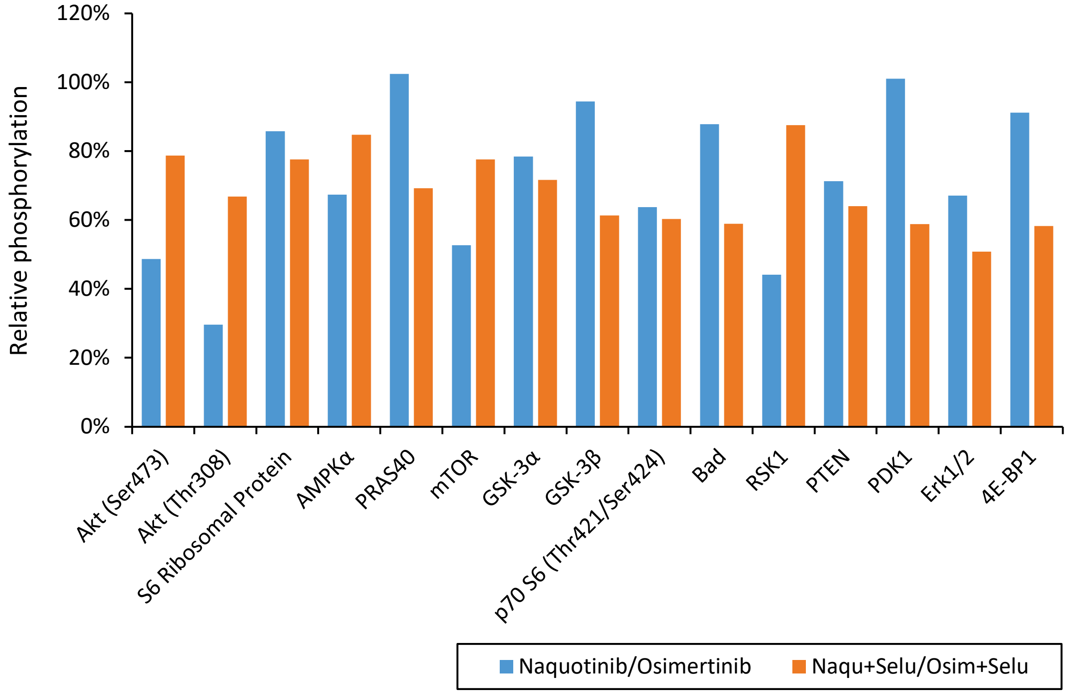

Supplementary Figure S10.
